# CD73: a new immune checkpoint for leukemia treatment

**DOI:** 10.3389/fimmu.2025.1486868

**Published:** 2025-03-06

**Authors:** Huan Gao, Tingting Zhang, Ke Li, Xia Li

**Affiliations:** ^1^ Marine College, Shandong University, Weihai, China; ^2^ Institute of Medicinal Biotechnology, Chinese Academy of Medical Sciences & Peking Union Medical College, Beijing, China

**Keywords:** CD73, CD39, leukemia, immune escape, immune checkpoint, clinical treatment

## Abstract

Recent studies on the pathogenesis of leukemia have led to remarkable advances in disease treatment. Numerous studies have shown the potential and viability of immune responses against leukemia. In the classical pathway, this process is often initiated by the upstream activity of CD39, which hydrolyzes extracellular adenosine triphosphate (ATP) and adenosine diphosphate (ADP) to AMP. Subsequently, CD73 acts on AMP to generate adenosine, contributing to an immunosuppressive microenvironment. However, CD73 can also utilize substrates derived from other molecules through the non-canonical NAD^+^ pathway, specifically via the CD38/CD203a/CD73 axis, further enhancing adenosine production and facilitating immune escape. Targeting CD73 has shown potential in disrupting these immunosuppressive pathways, thereby enhancing anti-leukemic immune responses and improving patient outcomes. Inhibiting CD73 not only reduces the levels of immunosuppressive adenosine but also increases the efficacy of existing immunotherapies, such as PD-1/PD-L1 inhibitors, making it a versatile therapeutic target in leukemia treatment. This review discusses the potential of CD73 as a therapeutic target and emphasizes its unique position in the immune escape mechanism of leukemia. Moreover, this review provides an overview of the current research progress and future trends, emphasizing the clinical significance of targeting CD73 and other potential therapeutic strategies in leukemia.

## 1 Introduction

Immune checkpoints (ICs) are immunosuppressive pathways that have evolved to prevent excessive immune responses and the overactivation of immune cells. Tumor cells can exploit these checkpoint molecules, evading immune surveillance and thereby promoting immune escape and accelerated metastasis. Given their high proliferation rate, malignant hematoma cells in the tumor microenvironment (TME) hasten the onset and progression of leukemia by fostering tumor cell immune escape. The multiple subtypes of leukemia pose a challenge in identifying new immune targets. CD73, emerging as a potential immune target for solid tumors, is increasingly being recognized to be vital in the occurrence and progression of leukemia. CD39 hydrolyzes adenosine triphosphate (ATP) and adenosine diphosphate(ADP) to adenosine 5′-monophosphate (AMP), while CD73 further converts AMP into adenosine (1, 2). Moreover, the non-classical CD38/CD203a/CD73 pathway also generates adenosine, which plays a significant role in maintaining the immunosuppressive TME (3, 4). Research indicates that adenosine concentration influences the progression of various leukemia types (5–9). Consequently, understanding the role of CD73 in leukemia can unveil novel treatment possibilities. This review seeks to elucidate the functional role of CD73 in leukemia by analyzing its structure, function, and expression in other solid tumors and immune environments, thereby shedding light on the developmental mechanisms of CD73 in leukemia.

## 2 Structure and function of CD73

Human CD73 is encoded by the *NT5E* gene, which resides between positions 14–21 on the long arm of chromosome 6 (10). Two post-transcriptional isoforms of CD73 have been identified. CD73, also known as extracellular 5′-nucleotidase, is a ribosidase encoded by *NT5E*, with a molecular weight of approximately 70 kD. Most CD73 molecules are anchored to membranes by glycosylphosphatidylinositol (GPI), metabolizing extracellular AMP into adenosine and inorganic phosphate (10, 11). A fraction of CD73 exists in the soluble form, exhibiting activities and functions partially akin to its membrane-anchored counterpart (12–14).

When GPI anchors are cut by using PI-PLC, soluble CD73 is released, usually with increased catalytic activity (15). Although soluble catalytic efficiency can be higher than that of anchored CD73, due to substrate transfer, utilization efficiency and the fact that TNF-a cannot only affect PLC, the actual total adenosine production is not as good as that of anchored CD73 (16). CD73 also has a form that is anchored by GPI in small extracellular vesicles (sEVs) (17), which are derived from CD8+T cells and play an important role in the regulation of the immune microenvironment (18). Both anchored and soluble forms of CD73 exhibit distinct expression patterns in various tissues and across species. CD73 shows varied tissue expression, predominantly in the colon, kidneys, brain, liver, heart, lungs, spleen, lymph nodes, and bone marrow. In the vascular system, CD73 primarily associates with vascular and lymphatic endothelia, playing a role in regulating leukocyte transport (19). Within the immune system, CD73 is found on the surfaces of macrophages, lymphocytes, regulatory T cells (Tregs), and dendritic cells (20). Analysis of CD73 expression in immune cells reveals higher relative expression in naïve B cells, naïve CD8^+^ T cells, and memory B cells, compared with lower expression in CD4^+^ T cells (21). CD73 expression in immune cells varies by species; in humans, it is expressed on most B cells and certain T cell subsets (22, 23); in mice, it is expressed predominantly in T cells (Tregs, natural killer [NK] cells) and some mature B cells (24–27). In normal human peripheral blood, CD73 expression is rare in T cells and is mainly found in B cells (28, 29). CD73 has different functions in different tissues and cells. For example, it mainly plays an immunomodulatory function on the surface of lymphocytes, including the activation and proliferation of lymphocytes and the adhesion process with endothelial cells. However, CD73 acts as an adhesion molecule in endothelial cells and promotes lymphocyte migration (30). CD73 exerts its functions through various mechanisms in the tumor microenvironment, inhibiting the activation and effector function of T cells, inhibiting the killing effect of NK cells, and promoting immunosuppressor cells (MDSCs and Tregs) through CD39/CD73/A2AR pathway (31–33). In addition, the CD73/CD39 adenosine production pathway has recently been shown to promote the expression of stemness (34) and EMT-related genes (35–37), maintain the immunosuppressor microenvironment, and promote tumor progression and metastasis. *NT5E*, a typical hypoxia-inducible factor (HIF) target gene, undergoes alterations in expression and function in hypoxic conditions (38).

Beyond hypoxia, various inflammatory mediators including transforming growth factor (TGF)-β (39), interferons [IFNs (40)], tumor necrosis factor (TNF) (41), interleukin (IL)-1β (42), and prostaglandin E2 (43) can upregulate the expression and function of CD73 (44, 45). Furthermore, the Wnt pathway and cyclic AMP signaling pathway as well as unsaturated fatty acids can regulate CD73 expression, indicating its regulation by multiple factors (46). CD73 also exists in post-translationally modified forms, including glycosylated (47) and ubiquitinated variants (48).

Structurally, CD73 is composed of two covalently linked subunits, each approximately 70 kD, and is anchored to the cell membrane via a GPI anchor site. The N-terminal domain of CD73 binds divalent Zn^2+^ and Co^2+^ for catalytic activity, whereas its C-terminal domain serves as a binding site for AMP (49). Functionally, CD73 is involved in two key extracellular metabolic pathways: AMP and nicotinamide adenine dinucleotide (NAD^+^) metabolism. In the AMP pathway, CD39 sequentially converts extracellular ATP and ADP into AMP, which is then dephosphorylated by CD73 to produce adenosine. Adenosine acts as a key regulator of immunosuppressive signaling by binding to specific adenosine receptors on immune cells, leading to the suppression of inflammatory responses. Adenosine interacts with specific G-protein–coupled receptors, such as A2AR, on T cells (50). Within the NAD^+^ pathway, the essential coenzyme NAD^+^ is released into the extracellular environment. During NAD^+^ metabolism, nicotinamide is converted to AMP by the ectonucleotide pyrophosphatase/phosphodiesterase family and subsequently to adenosine by CD73 (51). In addition to its role as a protease, CD73 functions as an adhesion molecule, facilitating the migration of both normal and tumor cells (52).

## 3 CD73 promotes immune-evasion mechanisms

**Figure 1:**
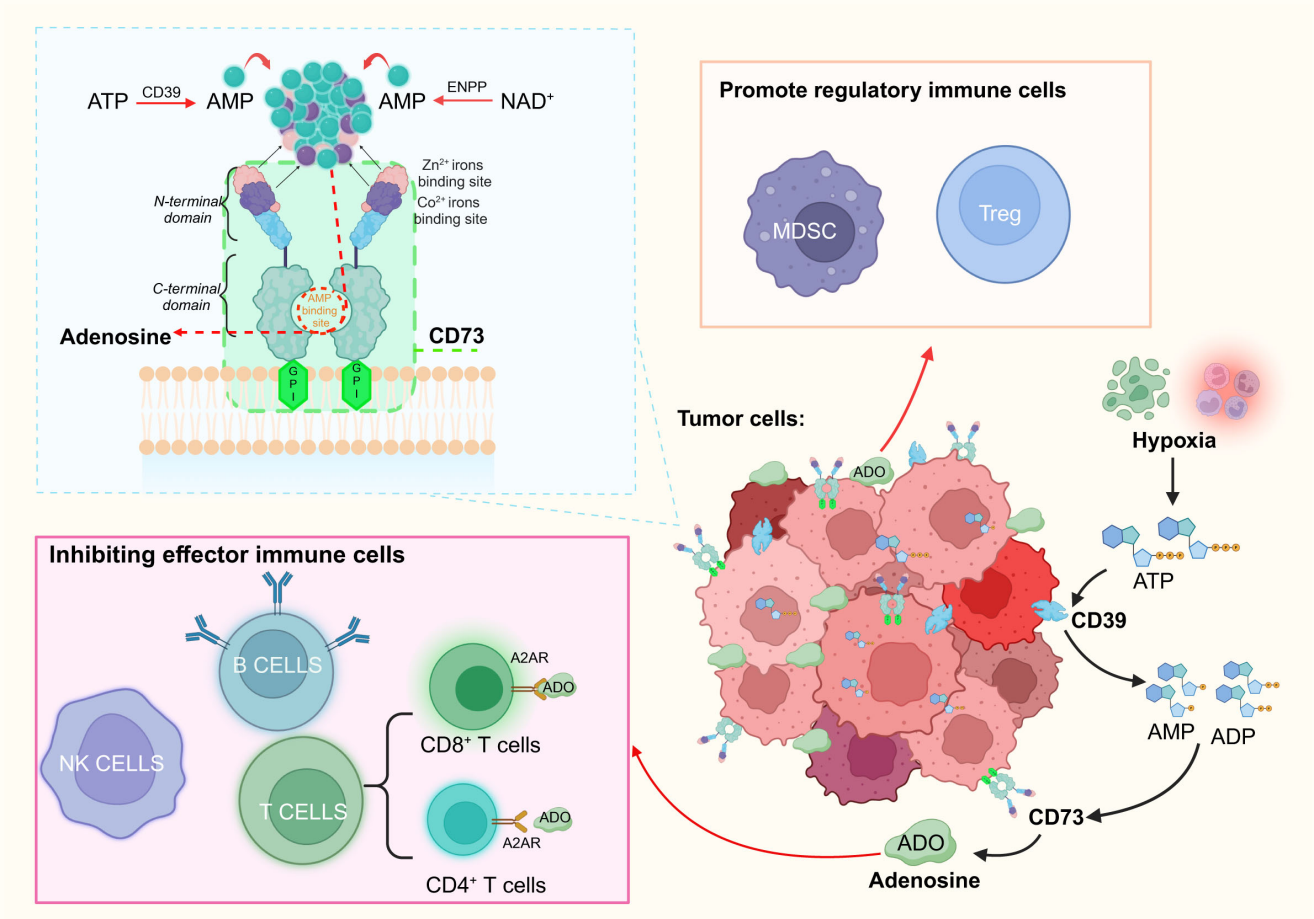
Schematic of the involvement of CD73 in the immune process through its structure and function, and the immune role of its product, adenosine, is briefly outlined. CD73, anchored to the membrane via GPI, is fully exposed on the exterior of the membrane. The C-terminal domain of CD73 specifically binds AMP to generate adenosine, whereas the N-terminal domain contains binding sites for Zn2+ and Co2+. Adenosine is produced via two primary pathways: the breakdown of ATP by CD39 and the NAD+ metabolism pathway. In tumor cells, the adenosine pathway facilitated by CD39/CD73 can suppress effector immune cells and bolster regulatory immune cells, thus sustaining the immunosuppressive tumor microenvironment. (Figure created with BioRender.com).

The role of CD73 expression in cancer is demonstrated in **Figure 1**, showing how it facilitates adenosine production and contributes to immune evasion. Numerous studies use CD73 as an IC marker for adenosine production, investigating its role in solid tumors and inflammation. In-depth research on the tumor immune microenvironment reveals that immune effector and regulatory cells, located at the tumor periphery, generate inflammatory responses to counteract cancer cell proliferation during immune infiltration (53). Similar to its role in the immunosuppressive TME, CD73 converts AMP into adenosine. Increased adenosine concentrations weaken the tumor-killing ability of immune effector cells (T cells, B cells, NK cells). In NK cells, adenosine mainly inhibits cytotoxicity of NK cells through A2AR signal, and mediates PKA to participate in tumor immune escape through CAMP-dependent signal (1, 54). In both CD4^+^T and CD8^+^T cells, adenosine binds to adenosine A2A receptor (A2AR), but inhibits the proliferation of T helper 1 (Th1) and T helper 2 (Th2) cells (55, 56) and promotes the differentiation of T helper 17 (Th17) cells after binding (57). A2AR is highly expressed in CD8^+^T central memory cells (TCM) in the tumor microenvironment, which is easily regulated by adenosine and leads to functional depletion of CD8+T cells (58, 59). Similarly, in B cells, adenosine exerts immunosuppressive effects through the activation of A2AR. Regulatory B cells expressing CD39 and CD73 produce adenosine. This increase in adenosine concentration weakens effector cell activity and enhances control by immune regulatory cells (Tregs, myeloid-derived suppressor cells), enabling tumor evasion from immune surveillance and attack. This shift transforms the immune microenvironment of the tumor into an immunosuppressive state.

Overexpressed CD73 in cancer cells results in elevated adenosine concentrations. Concurrently, cancer cell proliferation fosters a hypoxic environment, leading to lysis of normal cells and ATP production. ATP is converted into adenosine via the CD39/CD73 pathway, facilitating the immune escape process, which further enables cancer cells to metastasize and persist in immune evasion and proliferation (60). Factors such as HIF-1α are overexpressed in a hypoxic immune microenvironment, thereby aiding tumor immune evasion and promoting tumor growth. This process triggers the release of key cytokines, such as IFN-γ and IL-4, which modulate the immune response, leading to the suppression of T cell activation and proliferation. Their overexpression inhibits T cell activation and proliferation, adversely impacting the immune responses of cells (61). Programmed death 1 (PD1)/paradigm of surface-expressed programmed death ligand 1 (PDL1) is a popular pathway target in cancer research and has important therapeutic potential, which can reduce T cell activity through the interaction of PD1 and PDL1 (62–65). Studies have shown that both PD1/PDL1 and CD73/A2AR pathways (66) play a positive role in the negative regulation of immune response (67). Moreover, it has been confirmed that the CD39/CD73 pathway does not directly suppress PD1/PDL1 activity, but rather impairs the function of CD8+ T cells through increased adenosine production, contributing to resistance against anti-PD1 therapies (68).

CD73 expression varies significantly across cancer cell lines. For example, the leukemia cell line K562 does not express CD73, while high levels of CD73 are observed in glioblastoma (GBM) and commercial GBM cell lines such as U87 (69). In contrast, bladder cancer cells (70), melanoma cells (71), breast cancer cells (72, 73), lung cancer cells (74), pancreatic cancer (75), non-small cell lung cancer (76), cervical cancer cells (77), medulloblastoma cells (78), glioma cells (79) and ovarian cancer cell lines (80) also show varying levels of CD73 expression. In immune cells, CD73 plays a crucial role in modulating immune responses, particularly in natural killer (NK) cells, where it suppresses cytotoxic activity via the A2A receptor (A2AR) signaling pathway. This pathway facilitates tumor immune evasion by engaging cAMP-dependent mechanisms (81).

The role of CD73 in solid tumors is more intricate. In lung adenocarcinoma and non-small cell lung cancer (NSCLC), CD73 expression is linked to elevated PD-L1 levels and an increase in tumor-associated immune cells, contributing to an immunosuppressive microenvironment (74, 76, 82–86). Furthermore, CD73 has prognostic, oncogenic, and immunosuppressive roles in head and neck squamous cell carcinoma (87, 88). In hepatocellular carcinoma (HCC), CD73 activates the PI3K/AKT signaling pathway, leading to increased AKT phosphorylation and promoting tumor growth (89, 90). Moreover, CD73 is associated with immunosuppression and poor prognosis in pancreatic ductal adenocarcinoma (PDAC) (91–96). In thyroid and cervical cancers, TGF-β1 production promotes CD73 upregulation, contributing to cancer progression (97–100). In gastric cancer (GC), CD73 promotes immune evasion by impairing CD8+ T cell function (101, 102). Additionally, CD73/CD39+ cells have been implicated in the immunomodulation of chronic human immunodeficiency virus (HIV) infections (103, 104). High CD73 expression in invasive renal cell carcinoma is linked to increased cancer-related mortality (105). Moreover, CD73 has been identified as an important prognostic marker and a potential predictive biomarker for the efficacy of immunotherapy across various carcinomas. In gallbladder cancer, CD73, in conjunction with FcGBP, functions as a critical regulator of TGF-β1-induced epithelial-mesenchymal transition (EMT), a process strongly linked to tumor progression and poor survival outcomes (106). In cholangiocarcinoma, elevated CD73 expression acts as a prognostic biomarker, promoting EMT and correlating with decreased overall survival (107). Similarly, in colorectal cancer, high levels of CD73 are associated with a worse prognosis, reinforcing its role as a negative prognostic indicator in this malignancy (108). In melanoma, soluble CD73 serves as a biomarker for patients undergoing nivolumab therapy, highlighting its role in immune evasion and its utility as a predictive marker for immunotherapy response (109). Comprehensive analyses across multiple cancer types demonstrate that CD73 significantly influences the tumor microenvironment and immune response, establishing its importance as both a prognostic and therapeutic marker​ (110).

The potential of CD73 as a therapeutic target in several cancers, including breast and lung cancer, has shown promising therapeutic effects (111–113). In breast cancer, CD73 facilitates local invasion through the epidermal growth factor (EGF)/EGF receptor pathway (114–116). In addition, drug therapy studies in GBM have shown that while treatment increases CD73 expression, loss of CD73 significantly improves survival (117–120). This finding provides important clues for future anti-CD73 treatment strategies.

In summary, CD73 plays a pivotal role not only in inflammation by modulating immune responses through A2A receptor signaling but also in the progression of solid tumors by shaping an immunosuppressive microenvironment and activating oncogenic pathways. Furthermore, it exhibits potential as an immunotherapeutic target in the treatment of various subtypes of leukemia.

## 4 Role of CD73 in leukemia

Clinically, leukemia is often characterized by excessive white blood cells in the peripheral blood of patients (121). Accordingly, leukemia is classified into myeloid, lymphatic, chronic, and acute types (**Table 1**), and each classification has unique physiological characteristics (**Table 2**). The most common types of leukemia are acute myeloid leukemia (AML), acute leukemia/lymphoma (ALL), chronic lymphocytic leukemia/small lymphocytic lymphoma (CLL/SLL), and chronic myelogenous leukemia (CML). As white blood cells are highly mobile and encounter minimal obstruction, diseased white blood cells circulate throughout the body via the bloodstream. Consequently, the metastasis of leukemia involves a cyclic process that contributes to its high aggressiveness and fatality (122). Hematopoietic stem cells (HSCs) are developmentally superior to all lymphoid, myeloid, and hematopoietic cells (123), and mutations in HSCs or myeloid progenitor cells are linked to the development of AML and CML (124). Current research indicates that leukemia originates from single cells, with clonal evolution ensuing due to mutation accumulation (125, 126). In the blood of affected individuals, only certain leukemia cells, known as leukemia stem cells (LSCs), possess self-renewing stem cell properties. LSCs can induce disease when transplanted into a new immune-compatible host (127). Due to the resistance of LSCs to treatment (128), the disease evolves into phenotypically distinct subclones as mutations accumulate (129). Current studies have not yet clearly established the correlation between CLL, ALL, and LSCs. The complex nature of mutation accumulation complicates disease treatment and slows progress (130). The numerous subtypes of leukemia and the accumulation of inherited mutations contribute to the complexity of disease treatment.

**Table 1:** Types and characteristics of leukemia (121).

Leukemia Type	Key Characteristics	Prevalence	Typical Age of Onset
**Acute lymphoblastic leukemia (ALL)**	Fast-growing cancer of lymphocytes. Common in children; can occur in adults.	Most common type of cancer in children.	Mostly in children; can occur at any age.
**Acute myeloid leukemia (AML)**	Rapidly proliferating abnormal myeloid cells. Affects both children and adults.	Less common than ALL in children; more common in adults.	Primarily in adults; median age at diagnosis is 68 years.
**Chronic lymphocytic leukemia (CLL)**	Slow-growing cancer predominantly affecting B lymphocytes. Primarily seen in adults.	Most common type of leukemia in adults.	Mainly in adults > 50 years of age.
**Chronic myeloid leukemia (CML)**	Slowly progressing disease involving myeloid cells. Characterized by the Philadelphia chromosome.	Less common; affects adults.	Mainly in adults; median age at diagnosis is 64 years.

**Table 2:** Classification and Description of Leukemia Subtypes (https://www.cancer.net/).

Leukemia Type	Subtype	Description
**Acute myeloid leukemia (AML)**	AML with recurrent genetic abnormalities	AML characterized by specific chromosomal changes.
	AML with myelodysplasia-related changes	AML that evolves from previous myelodysplastic syndromes.
	AML with therapy-related myeloid neoplasms	AML resulting from previous chemotherapy or radiation.
	AML (NOS)	AMLs that do not fit into other categories.
**Acute lymphoblastic leukemia (ALL)**	B cell ALL	ALL affecting B lymphocytes with various genetic abnormalities.
	T cell ALL	ALL affecting T lymphocytes, including early T cell precursor lymphoblastic leukemia.
	Mixed lineage acute leukemias	Leukemias with features of both lymphocytic and myeloid types.
**Chronic lymphocytic leukemia (CLL)**	*IGHV*-mutated CLL	CLL with mutated *IGHV* gene; often with a better prognosis.
	*IGHV*-unmutated CLL	CLL without mutations in the *IGHV* gene; often more aggressive.
**Chronic myeloid leukemia (CML)**	Chronic phase	The initial phase of CML with fewer blast cells.
	Accelerated phase	CML with an increased number of blast cells, indicating progression.
	Blast crisis	The most advanced phase of CML with high blast cell count resembling acute leukemia.

**Figure 2:**
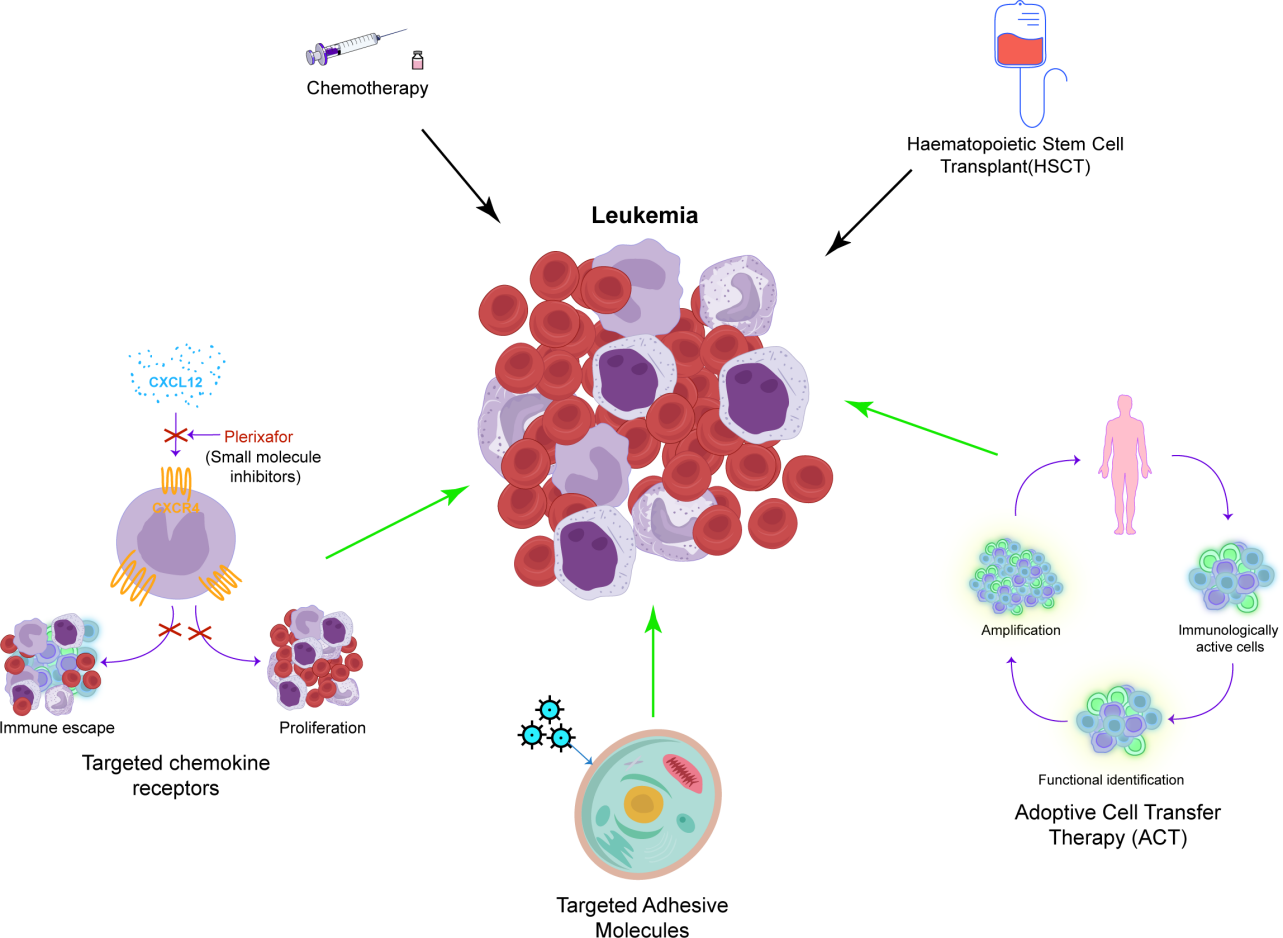
A concise overview of the recent approaches in leukemia treatment. This includes traditional methods such as chemotherapy and HSCT, as well as newer and specific techniques such as targeting chemokine receptors, adhesion molecules, and ACT.

Currently, chemotherapy is among the initial treatment strategies for leukemia (**Figure 2**) (131). While effective, its nonselectivity in targeting cells leads to significant adverse effects. Furthermore, the increasing resistance of leukemia cells to chemotherapy often results in its ineffectiveness and disease relapse (132). HSC transplantation (HSCT) involves the intravenous infusion of normal HSCs to restore the hematopoietic and immune functions in patients with leukemia who are undergoing induction therapy (133). HSCT is considered the most effective method to treat leukemia; however, it carries risks of severe rejection and immune disorders, and finding suitable donors poses a significant challenge (134).

Standard therapy is often ineffective in treating most types of leukemia in adults. Recent studies suggest that transforming acute leukemia into chronic leukemia can enhance survival rates by managing metastasis (122). Treatment methods include targeting the chemokine receptors, notably CXCR4, which are linked to leukemia cell proliferation and drug resistance (SDF1-CXCR4 axis). For instance, AMD3100, a specific CXCR4 antagonist, enhances the efficacy of chemotherapy by inhibiting SDF1-CXCR4, facilitating the entry of leukemia cells into the cell cycle (135). In the study of the SDF1-CXCR4 axis, effective therapeutic drugs such as monoclonal antibodies and novel small molecule inhibitors of CXCR4 have been discovered (136–139). The CD73 adenosine axis, which modulates the immune microenvironment by increasing adenosine production that suppresses antitumor immune responses, is emerging as a potential target for the treatment of leukemia. Additionally, targeting adhesion molecules such as selectins and integrins, which facilitate the lodging of leukemia cells within the bone marrow microenvironment, represents another promising approach. Inhibiting E-selectin, for example, disrupts pro-survival signaling in leukemia cells, thereby inhibiting AML stem cell regeneration and safeguarding native HSCs. GMI-1271, an E-selectin inhibitor, shows promising survival outcomes in the treatment of AML (140). Another target is the integrin family, particularly integrin α6. The BTK-integrin signaling pathway has shown positive results in preclinical studies (141–144), although further clinical validation is required (145). Adoptive cell therapy (ACT) is another option, wherein amplified immune cells such as NK cells, γδ T cells, and α-β T cells are transferred to patients for therapeutic outcomes. ACT can effectively delay the progression of leukemia, but challenges such as antigen selection and countering the immunosuppressive environment of tumors persist (146–150). Traditional treatments for leukemia include drugs that boost immune function to complement chemotherapy and radiotherapy. While these drugs may be effective against specific subtypes, issues such as immune system damage and drug resistance remain (151–153).

Recent studies on LSCs have identified targeted LSC proteins, IC molecules, and immune-related pathways. These studies aim to minimize adverse drug reactions and enhance treatment efficacy. However, this field is still in its developmental stages, thereby necessitating further research (154).

CD73, as an emerging IC, holds potential regulatory roles in the treatment of leukemia. Blocking CD73-related ICs in mouse models of CLL can effectively enhance immune responses. The study indicated that decreased CD73 levels reduced adenosine uptake by A2AR receptors, consequently lowering PD-1 expression (155). Mechanistically, PD-1 downregulation results in an increase in CD8^+^ T cells and enhanced IFN-γ secretion, thereby boosting immune response regulation and survival rates. Another study highlights that depleting CD8^+^ T cells enhances the migration, stemness, and proliferation of CLL cells (156). Additionally, CD8^+^ T cell depletion is linked to the CD73/adenosine axis in various cancer-related inflammation studies (157–159). High NT5E mRNA expression in samples from pediatric patients with B-ALL correlates with unfavorable clinical and pathological characteristics, abundant Tregs and dendritic cells, NK cell depletion, and elevated IC gene expression. However, the prognostic significance of CD73 at the protein level is not substantial (160). In addition to facilitating the immune escape of leukemia cells, CD73 also acts as a surface marker for different leukemia subtypes and supports leukemia cell proliferation. Flow cytometry revealed higher CD73 expression in samples from patients with B-ALL having minimal residual disease (MRD) than in those with MRD alone. Consequently, CD73 may serve as a marker for MRD in patients (161–163), although extensive studies are required for further conclusive evidence. NT5E drives the CEBPA transcription program in the CEBPA mutant subtype of AML. The tumor-promoting effect of CD73 may stem from CD73-dependent adenosine acting on A2AR to sustain leukemia cell growth and inhibit apoptosis (164). In AML, studies on bone marrow-derived mesenchymal stem cells (BM-MSCs) show CD73 as a cell surface marker and adhesion protein, similarly expressed in BM-MSCs (165). The ability of BM-MSCs to differentiate and support hematopoiesis *in vitro* suggests the involvement of CD73 in the progression of AML. In summary, CD73 is pivotal in inflammation and solid tumors and also exhibits potential as an immunotherapeutic agent in the treatment of various subtypes of leukemia. Studies on LSCs and MRD (166) suggest a significant relationship between CD73 and LSCs.

## 5 Expression regulation and post-translational modification of CD73

This section primarily discusses upstream transcription factors related to CD73, the impact of its post-translational modifications on function, and how these factors influence the progression of leukemia. The transcription factor HIF1-α and aryl hydrocarbon receptor (AHR) control metabolic program type 1 regulatory T cell (Tr1) differentiation. In hypoxia and inflammation, the inactivation of AHR by HIF-1α inhibits Tr1 differentiation, whereas ATP conversion by CD39 promotes its differentiation. CD73/adenosine involvement further inhibits Tr1 activity (167, 168). HIF1-α has recently been identified as a transcription factor that inhibits T cells and regulates the immunosuppressive TME through the CD39/CD73/A2AR/adenosine pathway (169–171). The Treg cell transcription factor Foxp3 suppresses immune function that is influenced by A2AR/adenosine (172). In mouse iNKT cells, IFN-γ production and A2AR are linked to inflammation and vascular damage, weakening A2AR function and reducing Th2-type cells, thereby diminishing immune function (173). Gene Ontology enrichment analysis of CD73 in breast tumors identified TGF-β and epithelial-mesenchymal transition (EMT) as significant inducers of CD73 expression (174), underscoring the role of HIF1-α in the cancer immune process. The transcription factor SNAI1 (part of the EMT pathway) upregulates CD73 expression in triple-negative breast cancer (116). Studies on melanoma reveal that MAPK signaling and the pro-inflammatory cytokine TNF-α co-induce CD73 expression via the c-Jun/AP-1 transcription factor complex, with IFN-γ also potentially having an impact on CD73 (175). In AML, increased CD39, PD-1, TIM-3, and LAG-3 expression in CD8T cells correlates with TNF-, IL-2–, and IFN-γ–induced decrease in CD73 protein expression (176). However, low CD73 protein expression suggests a link with CD8 T cell depletion. MicroRNA research on CD73 indicates that the regulatory factor miR-422a can directly or indirectly affect NT5E mRNA levels, influencing its binding to the transcription activator SMAD4 mRNA (177).

CD73 exhibits diverse forms and functions through post-translational protein modifications; it specifically undergoes two main modifications, namely, glycosylation and ubiquitination. Additionally, other forms such as lactate and hematoxylization modifications indirectly impact the functions of CD73. A study on chimeric antigen receptor T cell therapy for GBM showed that H3K18 lactate effectively enhanced the activity of *CD39*, *CD73*, and *CCR8* genes (178). Tumor metabolism–produced lactate upregulates CD39 and CD73 levels, increases the expression of CCR8 and its ligands, disrupts Treg/Th17 balance, and alters the immune microenvironment, demonstrating its role in promoting an immunosuppressive environment. A study on IκBα SUMO-1 modification in hypoxic environments has discussed the role of adenosine in SUMO-1 modification and NF-κB–mediated transcription (179), indicating potential targets for SUMOylation regulation of adenosine production and CD73 in the immune microenvironment. A study on CD73 glycosylation found that N-linked glycosylation selectively alters CD73 protein activity, leading to high mannose glycosylation and enzymatically impaired glycosylation in HCC (47). Additionally, the study showed that glycosylation might impair both CD73 expression and its normal enzymatic activity. A study on cervical cancer revealed that highly glycosylated, soluble CD73 increased AMP activity (180), contrasting previous findings and suggesting functional differences between membrane-bound and soluble CD73 after glycosylation. Recent studies have identified TRIM21 as the E3 ubiquitin ligase that directly targets CD73. Knocking down TRIM21 in breast cancer cells leads to CD73 overexpression, thereby promoting cancer progression (48). TRIM21 regulation, influenced by T cell–secreted IFN-γ, helps maintain a stable adenosine microenvironment in normal cell lines. TRIM21 overexpression can result in reduced CD73 expression and improved prognosis. The study highlights a novel immunotherapeutic target acting directly on CD73 and offers insights for future ubiquitination research in CD73-related cancer cell lines. Overall, research on post-translational modification of CD73 is limited, particularly CD73 modification in leukemia. Considering that most existing studies focus on post-translational modifications and CD73, this area holds significant potential in leukemia research.

## 6 Significance of targeting CD73 in the treatment of leukemia

With progress in CD73 research, several drugs targeting CD73 have been developed, with some candidates advancing to clinical trials (**Table 3**). CPI-006, a humanized immunoglobulin (Ig)G1 FcγR-binding defective antibody, specifically binds to CD73^+^ T and B lymphocytes by targeting the N-terminus of CD73. It possesses unique characteristics as a CD73 inhibitor. The binding reduces the catalytic activity of CD73 and enhances immune-regulatory functions (181). CPI-006 is currently in phase 1 clinical trials and has demonstrated efficacy in reducing B cell count by binding to CD73 in 34 patients with advanced cancer (181). AB680, another efficacious CD73 inhibitor, exhibits reversible and selective binding to CD73 and is currently in phase 1 clinical trials (182). AB680 significantly enhances CD8^+^ T cell infiltration and prolongs survival of mice (183). TJ004309, a monoclonal antibody targeting CD73, completely inhibits CD73 activity and reduces adenosine production by binding to CD73 and is now in phase 1 to 2 clinical trials (184). HLX23, a recombinant anti-CD73 humanized monoclonal antibody, is in phase 1 clinical trials for the treatment of advanced solid tumors. Oleclumab, a selective anti-CD73 monoclonal antibody, is effective in treating advanced solid tumors, especially in combination with durvalumab (185). It is currently in phase 2 clinical trials (https://clinicaltrials.gov/). AK119, a humanized IgG1 monoclonal antibody, selectively binds to and inhibits the exonucleotidase activity of CD73 (186). AK104 is a recombinant humanized IgG1 bispecific antibody targeting PD-1 and CTLA-4 simultaneously. While CD73 blockade therapy is popular in treating various cancers, CD73 and PD-1 therapies alone often yield unsatisfactory results, possibly due to the nonoverlapping, immunosuppressive mechanisms of tumors in immune escape, including adenosine accumulation (187). The inhibitory effect of CD73 monoclonal antibodies varies with different concentrations and binding sites (188). Controlling drug concentration and drug targeting *in vivo* is more challenging. The combination of drugs targeting both the CD73/adenosine pathway and PD-1/PDL1 is gaining popularity owing to their crucial roles in immune escape and the limited effects of either therapy alone. As CD73 emerges as a key immune target in tumor research, the development of monoclonal antibodies targeting CD73 is advancing. As CD73 is being recognized as an emerging immune target in tumor therapy, several treatments including monoclonal antibodies, small molecule inhibitors, and drug combinations with other immune pathways are being developed, with clinical research advancing rapidly. However, challenges in significantly enhancing cancer treatment persist due to unclear mechanisms of nonoverlapping immune suppression in cancer and current treatment outcomes (189). This remains true despite ongoing advancements in combination therapy and CD73 monoclonal antibodies. Consequently, using targeted research, there is a need to identify new targets for the immune escape process of cancers and to develop more specific and stable monoclonal antibodies as well as small molecule inhibitors.

**Table 3:** Current studies targeting CD73 target therapy and combination therapy (https://clinicaltrials.gov/).

Drug	Company	Phase	NCT Number
CPI-006| CPI-006 + Ciforadenant| CPI-006 + Pembrolizumab| CPI-006| CPI-006 + AK119	Corvus Pharmaceuticals, Inc.	PHASE1	NCT03454451
	Akeso	PHASE1	NCT05173792
AB680| Zimberelimab| Nab-paclitaxel| Gemcitabine	Arcus Biosciences, Inc.	PHASE1	NCT04104672
Durvalumab, Tremelilumab, MEDI 9447, MEDI 0562	Nordic Society of Gynaecological Oncology	PHASE2	NCT03267589
TJ004309| Atezolizumab	Tracon Pharmaceuticals Inc.	PHASE1	NCT03835949
TJ004309| Toripalimab	I-Mab Biopharma Co. Ltd.	PHASE1| PHASE2	NCT04322006
Durvalumab| Oleclumab	University Health Network, Toronto	PHASE2	NCT06060405
HLX23	Shanghai Henlius Biotech	PHASE1	NCT04797468
CD73 Antigen	Biotheus Inc.	PHASE1	NCT05950815
Dalutrafusp alfa| mFOLFOX6 Regimen| Dalutrafusp alfa	Gilead Sciences	PHASE1	NCT03954704
Domvanalimab| Quemliclustat| Zimberelimab| Fluorouracil| Leucovorin| Oxaliplatin	Arcus Biosciences, Inc.	PHASE2	NCT05329766
AB680| Etrumadenant| Zimberelimab| Bevacizumab| m-FOLFOX-6 Regimen | Regorafenib	Arcus Biosciences, Inc.	PHASE1| PHASE2	NCT04660812
TJ004309	I-Mab Biopharma US Limited	PHASE2	NCT05001347
IBI363| IBI363| IBI363| IBI363| IBI363	Hunan Province Tumor Hospital	PHASE1	NCT06081907
LY3475070| Pembrolizumab	Eli Lilly and Company	PHASE1	NCT04148937
AK131	Akeso	PHASE1	NCT06166888
IBI325 + Sintilimab| IBI325	Innovent Biologics (Suzhou) Co. Ltd.	PHASE1	NCT05119998
IBI325 + Sintilimab	Shandong Cancer Hospital and Institute	PHASE1	NCT05246995
ORIC-533	ORIC Pharmaceuticals	PHASE1	NCT05227144
Oleclumab| Durvalumab	MedImmune LLC	PHASE1	NCT02503774
PT199| Anti-PD-1 Monoclonal Antibody	Phanes Therapeutics	PHASE1	NCT05431270
Osimertinib	Nantes University Hospital	PHASE2	NCT03865511
IPH5301| CHEMOTHERAPY AND TRASTUZUMAB	Institut Paoli-Calmettes	PHASE1	NCT05143970
Etrumadenant| Zimberelimab| Quemliclustat| Enzalutamide| Docetaxel| SG	Arcus Biosciences, Inc.	PHASE1| PHASE2	NCT04381832
AGEN1423| Balstilimab| Gemcitabine| Nab-paclitaxel	Bruno Bockorny, MD	PHASE2	NCT05632328
Sym021| Sym024	Symphogen A/S	PHASE1	NCT04672434
Durvalumab| Oleclumab	Jules Bordet Institute	PHASE2	NCT03875573
AK119| AK112	Akeso	PHASE1| PHASE2	NCT05689853
HB0045	Shanghai Huaota Biopharmaceutical Co., Ltd.	PHASE1| PHASE2	NCT06056323
S095018| S095024| S095029| S095018| S095024RDE| S095029RDE| Cemiplimab	Servier Bio-Innovation LLC	PHASE1| PHASE2	NCT06162572
AK119| AK104	Akeso	PHASE1| PHASE2	NCT05559541

Despite the therapeutic promise of CD73 inhibitors, potential side effects and limitations require careful consideration. Recent murine studies demonstrate that CD73 inhibition enhances bone marrow stem cell mobilization, potentially disrupting hematopoietic homeostasis and increasing risks of myelopathic suppression or accidental stem cell dissemination (190). Notably, since adenosine maintains critical physiological functions including cardiovascular regulation and neuroprotection (191, 192) systemic CD73 blockade may compromise these protective mechanisms. Furthermore, therapeutic efficacy could be limited by compensatory upregulation of alternative adenosine-producing pathways [such as the CD38/CD203a axis (3)] or the emergence of resistance mechanisms within the tumor microenvironment.

Currently, publicly available data on the use of CD73 in treating leukemia are limited. A noteworthy study at the University Hospital of Tours in France (ClinicalTrials.gov ID NCT05792007) focused on the medullary microenvironment of acute childhood leukemia and explored the energy metabolism in mesenchymal stem cells, including oxidative phosphorylation and glycolysis. CD73 is anticipated to be a promising anticancer target in this study. Currently, the mechanism of effectiveness of CD73 has not yet been well established and the underlying reasons are unclear. One possibility is that drug resistance of cancer cells contributes to the diminished efficacy of CD73 and PD-L1–targeted drugs, resulting in recurrence. However, targeting CD73 in leukemia treatment still holds significant potential. Studies indicate that CD73 is present in patients with MRD after recovery (162, 163, 193) and is likely linked to leukemia HSCs. Additionally, during treatment with CD73, potential changes in the modified functional structure of CD73 that affect adenosine production and drug specificity must be considered.

## 7 Future research directions related to CD73 in leukemia

First, studies of solid tumors indicate the multiple potential functions of CD73 and that soluble CD73 can influence the immune microenvironment. These findings differ from the common belief that the primary function of CD73 in normal cells is membrane anchoring. Second, the mechanism of transcription factors in regulating CD73 expression remains unclear and most factors that affect CD73 do so indirectly. Transcription factors modulate the function of CD73 by influencing the functional structure of other proteins. Research on transcription factor targets of CD73 is incomplete and there is no specific transcription factor that has been identified to directly target CD73. Therefore, exploring the transcription factor targets of CD73 could be a promising avenue for further in-depth research. Beyond transcription factors, the growing focus on post-translational modifications has highlighted the importance in protein function, particularly for enzymes such as CD73. Our current understanding of how post-translational modifications regulate CD73 and their impact on the function of CD73 is incomplete, and the discovery of soluble glycosylation of CD73 (180) underscores the substantial impact of this modification on its function. Given the varied functional mechanisms of CD73 in different immune tumor microenvironments, investigating its modification changes and functional effects across various solid and hematological cancers is essential. The discovery of TRIM21 as an E3 ubiquitin ligase suggests CD73, potentially influenced by TRIM21 and other modifiers, to be a promising therapeutic target less susceptible to drug resistance.

A major challenge in studying the role of CD73 in leukemia is analyzing its regulatory effect on the disease through expression in isolated LSCs and identifying its action sites using techniques such as lineage tracing. It is also crucial to investigate whether post-translational modifications affect the structure of CD73 so that its post-denaturation involvement can be ascertained. Exploring the role of CD73 in leukemia is just the beginning; subsequent research should focus on designing small molecule inhibitors or monoclonal antibodies that target CD73 expression at specific sites, which is informed by its role in the immune evasion of leukemia and drug resistance mechanisms.

While CD73 is a promising target in the treatment of leukemia, significant challenges arise from its complexity and the various subtypes of leukemia. Ongoing research on CD73 in leukemia suggests a strong correlation between CD73-involved signaling pathways and different leukemia subtypes. Additionally, protein modification research implies that CD73 mRNA expression surpasses protein expression because some of the expressed CD73 proteins may have lost their function. The expressed CD73 often concentrates in small residues, akin to the characteristics of LSCs, which suggests that CD73 might be a key marker of LSCs, holding significant treatment implications for leukemia. Considerable research related to drug screening needs to be further undertaken. There is not only a need for highly specific and stable monoclonal antibodies and small molecule inhibitors but also investigations of the additional nonoverlapping immunosuppressive mechanisms akin to the CD73/adenosine and PD1/PDL1 pathways to enhance the survival rates of patients with leukemia and other cancers.
